# Supplementary figures and images for: Beyond GWAS—Could Genetic Differentiation within the Allograft Rejection Pathway Shape Natural Immunity to COVID-19?
Source: Int J Mol Sci. 2022 Jun 3;23(11):6272. doi: 10.3390/ijms23116272 (PMC9181155; doi:10.3390/ijms23116272)

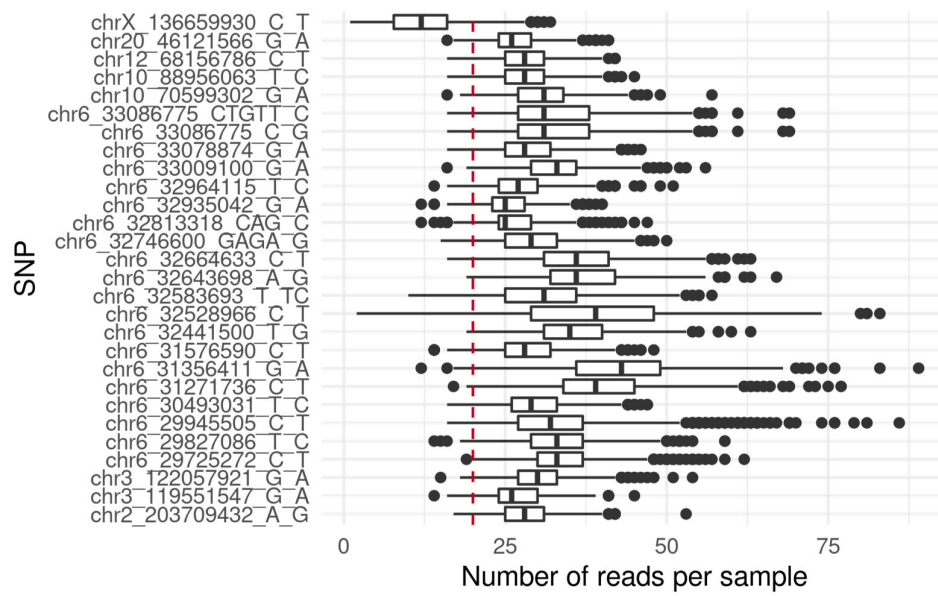

Figure S1. Number of reads per sample.

Supplement: Supplementary file 1 [file ijms-23-06272-s001.zip › ijms-1711701-SM.pdf]
